# Status of insecticide resistance in *Anopheles* mosquitoes in Ubon Ratchathani province, Northeastern Thailand

**DOI:** 10.1186/s12936-017-1948-z

**Published:** 2017-07-25

**Authors:** Anchana Sumarnrote, Hans J. Overgaard, Nattapol Marasri, Bénédicte Fustec, Kanutcharee Thanispong, Theeraphap Chareonviriyaphap, Vincent Corbel

**Affiliations:** 1 Department of Entomology, Faculty of Agriculture, Kasetsart University, Bangkok, Thailand; 2 Bureau of Vector-borne Disease, Department of Disease Control, Ministry of Public Health, Nonthaburi, Thailand; 3 Maladies Infectieuses et Vecteurs, Ecologie, Génétique, Evolution et Contrôle (MIVEGEC), Institut de Recherche pour le Développement (IRD), Montpellier, France; 4 Department of Mathematical Sciences and Technology, Norwegian University of Life Sciences, Ås, Norway

**Keywords:** Malaria, Vectors, *Anopheles*, Pyrethroids, DDT, Resistance, Synergists, kdr

## Abstract

**Background:**

Malaria is common in hilly, forested areas along national borders in Southeast Asia. Insecticide resistance in malaria vectors has been detected in a few countries in the Greater Mekong sub-region (GMS), representing a threat to malaria control and prevention. This study aims to determine the insecticide resistance status of *Anopheles* mosquitoes in Ubon Ratchathani province, northeastern Thailand, where increasing number of malaria cases were reported recently.

**Methods:**

Mosquitoes were collected in 2013–2015 using human landing and cattle bait collections in six sites during both the rainy and dry seasons. Mosquitoes were first morphologically identified to species and their susceptibility status to deltamethrin (0.05%), permethrin (0.75%) and DDT (4%) investigated, according to WHO guidelines. Bioassays with the synergists PBO and DEF were carried out to address the role of detoxifying enzymes in insecticide resistance. DNA sequencing of a fragment of the voltage-gated sodium channel gene was carried out to detect knock-down resistance (*kdr*) substitutions at position 1014 in resistant species.

**Results:**

Due to low vector abundance, complete bioassays (n ≥ 100 mosquitoes) were only achieved for *Anopheles hyrcanus* s.l., which was resistant to all insecticides tested (mortality ranged from 45 to 87%). Suspected resistance to DDT was found in *Anopheles barbirostris* s.l. (mortality 69%), but it was susceptible to deltamethrin (mortality 97–100%) and permethrin (mortality 100%). Although insufficient number of primary vectors were collected, results showed that *Anopheles dirus* s.l. and *Anopheles maculatus* s.l. were susceptible to deltamethrin (mortality 100%)*. Anopheles nivipes* and *Anopheles philippinensis* were susceptible to all three insecticides. PBO significantly increased mortality to deltamethrin and permethrin in pyrethroid-resistant *An. hyrcanus* s.l. None of the sequenced specimens presented the L1014F or L1014S mutation.

**Discussion:**

This study shows that insecticide resistance is present in potential malaria vectors in northeastern Thailand. The absence of *kdr* mutations in all *Anopheles* species tested suggests that metabolic resistance is the main mechanism of pyrethroid resistance. This study provides new findings about insecticide susceptibility status of potential malaria vectors in northeastern Thailand that are deemed important to guide malaria vector control.

## Background

In 2015, an estimated 3.2 billion people in 97 countries were at risk of malaria [1]. In the Greater Mekong sub-region (GMS), malaria foci are located in forested and rural areas and along country borders [2]. From these foci, malaria can spread to currently malaria free areas [3, 4]. The important economic and social implications caused by malaria in the region have prompted governments to make this disease a public health priority and to implement integrated national malaria control programs adapted to the specific needs of their individual countries [5]. In Thailand, the number of malaria cases has decreased substantially since the 2000s, but the disease remains a major cause of morbidity for people living in border areas (i.e. about 32 million people) [6].

Vector control has played an essential role in the reduction of malaria in Thailand and the country has entered into the pre-elimination phase [7]. At present, vector control relies mainly on indoor residual spraying (IRS) and insecticide-treated nets (ITNs). Indoor residual spraying with deltamethrin once or twice a year is applied following the guidelines and malaria stratification system of the national malaria control programme [8]. According to the 2015 annual malaria report of the Bureau of Vector Borne Disease (BVBD), bifenthrin and alpha-cypermethrin have also been used for IRS, whereas permethrin and deltamethrin are the two main pyrethroids used for ITNs [8].

Unfortunately, the repeated use of the same insecticides in public health and for agricultural purposes has contributed to selection for insecticide resistance in malaria vector populations worldwide [5, 9, 10]. Although the situation is less worrying in South East Asia than in Africa [9], insecticide resistance is on the rise and may pose increasing challenges to malaria control and elimination. The World Health Organization (WHO) recommends member countries to implement an active system of insecticide resistance monitoring of vectors to improve preventive strategies and achieve malaria elimination [11]. A Global Plan for Insecticide Resistance Management (GPIRM) has been developed and was released in May 2012 by the WHO. The strategies of IRM aim to preserve insecticide susceptibility, slow down the spread of insecticide resistance when its already present and develop new approaches for sustainable vector control [12]. All countries in the GMS have set national malaria elimination goals to eliminate malaria by 2030 [7].

In the GMS, Van Bortel et al. previously reported resistance to lambda-cyhalothrin and suspected resistance to alpha-cypermethrin in *Anopheles dirus* sensu stricto (s.s.) in central Vietnam. In the Mekong delta, *Anopheles epiroticus* was resistant to all pyrethroid insecticides tested whereas suspected resistance to DDT was only found in Ho Chi Minh City [5]. The same authors reported *Anopheles minimus* sensu lato (s.l.) populations being resistant to pyrethroids in North Vietnam, but susceptible in Cambodia, Lao PDR and Thailand. Two *An. minimus* s.l. populations showed DDT resistance in western Cambodia and Northern Vietnam and *Anopheles vagus* was found to be highly resistant to DDT and pyrethroids in Vietnam and Cambodia [5].

Insecticide resistance is a heritable trait that occurs in a mosquito population by migration (i.e. introduction of resistance genes in a new population) or de novo mutation. Once resistance is present, continual exposure of mosquitoes (larvae or adult) to insecticides will increase the frequency of individuals having the resistance trait until the gene become fixed. Through this process of selection, the population gradually acquires strong resistance to the insecticide. The dispersal capabilities of mosquitoes can also act as a primary factor of resistance development and dispersal. In contrast, movement of mosquito populations between treated and untreated areas (refuge zones) can delay the development of resistance by dilution of resistant pools by susceptible immigrants.

The mechanisms of insecticide resistance in mosquitoes can be classified into two broad categories including physiological change and behavioral avoidance. Physiological resistance has been shown to involve three factors, i.e., reduced penetration of insecticides trough the cuticle; metabolic resistance, i.e. presence of enzymes that detoxify the insecticide; and target site resistance, i.e. prevention of the insecticide binding or interacting at its site of action. The major mechanisms of insecticide resistance involve either mutations within the target site of the insecticide and/or an alteration in the rate of insecticide detoxification [13]. The voltage-gated sodium channel protein is the major target for both pyrethroids and DDT. Many studies have reported that two substitutions at codon 1014 of the voltage-gated sodium channel (VGSC) are associated with knockdown resistance [14–16]. A leucine-to-phenylalanine mutation at residue 1014 is the most common mutation in insects including anophelines [17]. In the GMS, the 1014S *kdr* mutation was reported in Vietnam, Cambodia and Lao PDR in *An. vagus*, *Anopheles sinensis* and *Anopheles paraliae* populations resistant to pyrethroids and DDT [15]. In *Anopheles peditaeniatus*, both 1014F and a 1014S *kdr* alleles were found in southern Vietnam [15]. In Korea, a leucine-to-cysteine (L1014C) substitution was found in *An. sinensis* populations exhibiting resistance to permethrin [18]. Metabolic resistance, cytochrome P450 monooxygenases (P450s) are enzymes known to metabolize a wide variety of compounds in mosquitoes including insecticides [13]. In Thailand, increased mRNA expression of two P450 genes, CYP6P7 and CYP6AA3, suspected to metabolize some pyrethroids [19], have been found in a deltamethrin-resistant population of *An. minimus* [20, 21].

Between 2000 and 2010, resistance to DDT was detected in *Anopheles annularis* and *An. minimus* located in the northwestern part of the country [22]. The primary vectors, *An. dirus* s.l., *An. minimus* s.l., and *Anopheles maculatus* s.l., showed to be mostly susceptible to insecticides, except in the northern part of the country (Chang Mai province) where deltamethrin, cyfluthrin, and malathion resistance was detected in *An. minimus* s.l. [4, 21].

Ubon Ratchathani province is located in the northeast of Thailand along the Cambodia and Lao PDR borders. In 2014, there was a malaria outbreak in the province, recording the highest number of malaria cases compared to all other provinces [23]. The majority of malaria cases are consistently being reported from four districts in the province, namely Buntharik, Nachaluay, Nam Yuen and Khong Chiam [23]. Successful implementation of malaria vector control strategies in Ubon Ratchathani requires recent information on the status of insecticide resistance in *Anopheles* mosquitoes. There is a paucity of data on resistance to insecticides conventionally used for malaria vector control in this region. The current study aimed at characterizing the status and mechanisms of insecticide resistance to public health insecticides in *Anopheles* mosquitoes to guide malaria vector control.

## Methods

### Study site

**Fig. 1:**
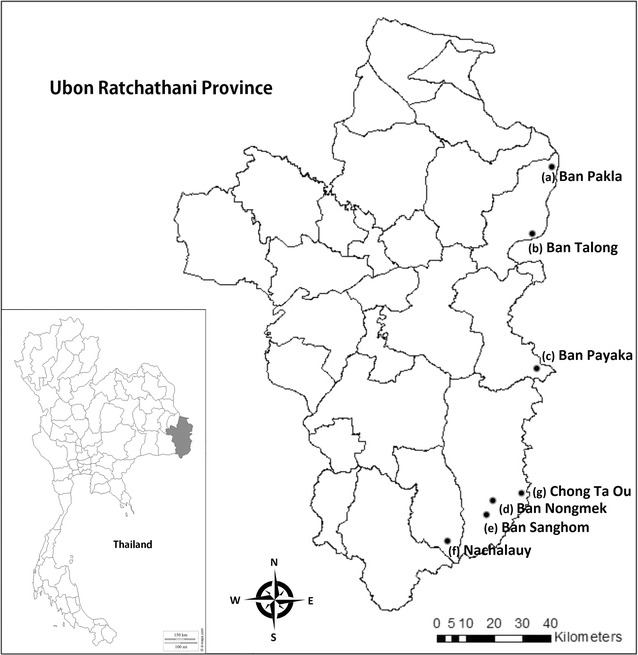
The collection sites located along on the Thailand–Laos border: **a** Ban Pakla (Khong Chiam district), **b** Ban Talong (Khong Chiam district), **c** Ban Payaka (Sirindhorn district), **d** Ban Nongmek (Buntharik district), **e** Ban Sanghom (Buntharik district), **f** Rubber plantation in Nachaluay district and **g** Chong Ta Ou (Buntharik district)

Mosquitoes were collected from seven sites in five districts in Ubon Ratchathani province: (a) Ban Pakla, Khong Chiam district (15°38.822′N–105°37.968′E), (b) Ban Talong, Khong Chiam district (15°24.313′N–105°33.801′E), (c) Ban Payaka, Sirindhorn district (14°58.821′N–105°31.097′E), (d) Ban Nongmek, Buntharik district (14°35.454′N–105°22.505′E), (e) Ban Sanghom, Buntharik district (14°34.578′N–105°21.615′E), (f) a rubber plantation in Nachalauy district; and (g) Chong Ta Ou, a border patrol police camp in Buntharik district (14°42.309′N–105°31.053′E) (Fig. 1). These sites were selected based on the high prevalence of malaria and being located near the Lao border with high risk factors for infection, such as presence of forests, high-risk occupations and poor knowledge prevention practice [23]. Additional mosquito collections were carried out in September 2015 to strengthen the effort to collect specific forest-associated malaria vectors, particularly *An. dirus* and to assess metabolic resistance mechanisms in *An. hyrcanus* by bioassays using PBO and DEF synergists. The additional collections were done in four locations: (1) Chong Ta Ou, a border police station close to the Lao border, surrounded by mixed deciduous forest; (2) Ban Pakla, near the collection houses used in the regular collections and close to dry dipterocarp forests; (3) Ban Nongmek; and (4) Ban Sanghom. Mosquitoes collected in the last two sites were used to assess metabolic resistance mechanisms by using synergists. Residents living in these areas are mainly farmers, involved in rice production, rubber, forestry and non-wood forest product collection.

### Mosquito collections

Between September 2013 and September 2015, five entomological surveys were carried out. Mosquito collections were carried out twice a year during both the dry and rainy seasons using indoor and outdoor human-landing (HLC) and cow bait collections (CBC) [24] (Table 1). In each village, one site was used for the CBC and four houses were used for HLC (both inside and outside the same house). Collections were carried out from 06.00 p.m. to 06.00 a.m. for four consecutive nights per survey (equivalent to 16 person-nights collections per village for HLC and 4 cow-nights collections per village for CBC). The HLC lasted for 45 min each hour followed by a 15 min break for collectors. Human catch sites were located minimum 30 m from each other to avoid potential bias in attracting mosquitoes. The CBC involved placing a single adult cow inside an enclosure made of clean (untreated) cotton bed net measuring approximately 20 m in length and 1.5 m in height with the net suspended 30 cm above the ground level to allow mosquito access [25]. The cow net was placed approximately 100 m from the nearest HLC to avoid interference in mosquito attractiveness. The cow was exposed to mosquitoes entering the net uninterrupted for 45 min each hour, and then mosquitoes were collected for 15 min every hour by a collector using an aspirator. Mosquitoes landing on humans or resting on the net, at time of collection, were caught individually by aspirators or glass tubes. Collected mosquitoes were kept in a clean plastic cup covered with netting and provided with 10% sugar solution soaked on cotton. Cups were then brought back to the field station for further processing. All collection sites were geo-referenced using a Global Positioning System unit.

**Table 1:** Mosquito collection times in the seven locations in Ubon Ratchathani province, Thailand

Villages	Sub-district	District	Collection time				
			Rainy Sep–Oct 2013	Dry Mar 2014	Rainy Sep–Oct 2014	Dry Mar 2015	Rainy Sep 2015
Ban Pakla	Na Pho Klang	Khong Chiam	X	X	X	X	X
Ban Talong	Huai Pai	Khong Chiam	X	X	X	X	–
Ban Payaka	Non Ko	Sirindhorn	X	X	–	–	–
Ban Nongmek	Huai Kha	Buntharik	X	X	X	X	X
Ban Sanghom	Huai Kha	Buntharik	X	X	X	X	X
Rubber plantation	Nachaluay	Nachaluay	–	–	X	X	–
Chong Ta Ou	Phon Ngam	Buntharik	–	–	–	–	X

### Species identification

All female *Anopheles* mosquitoes collected were morphologically identified to species or species complex using stereomicroscopes and morphological keys [26]. Mosquitoes were then separated by species group, complex or species for bioassays. Mosquitoes were kept alive by providing sugar solution.

### Insecticide susceptibility tests

Bioassays were performed on adult mosquitoes using the standard WHO susceptibility bioassay test with diagnostic concentrations of DDT (4%), deltamethrin (0.05%), and permethrin (0.75%) [27]. The following species, *An. hyrcanus* s.l., *An. barbirostris* s.l., *Anopheles philippinensis*, *Anopheles nivipes*, *An. dirus* s.l., *An. maculatus* s.l., and *An. vagus* were subjected to bioassays the next morning after the night collection [27]. Adults could not always be collected in appropriate numbers (minimum 100) for bioassays; therefore, tests were done over several days. Mosquitoes were exposed for 60 min in tubes placed in vertical position. All control and insecticide-impregnated papers were supplied by the Vector Control Research Unit, Universiti Sains Malaysia and were not used more than four times. During exposure, the number of knocked-down mosquitoes was recorded every 5 min until 60 min. After 1 h exposure, mosquitoes were put in plastic cups with sugar solution and maintained for 24 h. The number of knocked-down mosquitoes and percentage mortality after 24 h were recorded. The susceptibility tests were carried out at 24–33 °C and 52–85% relative humidity. Mosquito samples were stored individually in 1.5 ml microtubes with silica gel and kept at −20 °C for molecular tests.

### Synergist bioassays

In order to explore the involvement of detoxifying enzymes in the resistant phenotype, synergist bioassays were performed when sufficient number of mosquitoes could be collected (September 2015). Two synergists, 0.25% S.S.S-tributyl phosphotritioate (DEF), an inhibitor of esterases and 4% pyperonyl butoxide (PBO), an inhibitor of oxidases were used in this study [27]. Different concentrations of synergists have been tested in various field studies, for example a 4% PBO concentration was used to test a range of *Anopheles* species in Sri Lanka [28], and 4% PBO and 0.25% DEF were used on *Anopheles gambiae* s.l. in Cameroon [29]. Experiments were done in the Entomology Lab at Kasetsart University using these concentrations to establish whether the same minimum concentrations could be used for Southeast Asian mosquitoes. The same concentrations of each synergist were assessed to be suitable, since they did not kill a laboratory strain of *Anopheles minimus* after 24 h (sub-lethal dose). The impregnation of the papers was performed at the Department of Entomology, Kasetsart University. Rectangular pieces of filter-paper measuring 12 × 15 cm (Whatman^®^ No. 1) were impregnated according to the WHO protocol using 2 ml of acetone solvent mixed with the non-volatile carrier silicon oil. During the assay in the field, mosquitoes were first exposed 1 h to synergist papers before being exposed 1 h to the insecticide.

### Extraction of mosquito DNA

Mosquitoes were put in 1.5 ml microcentrifuge tubes with two grinding balls. Disruption was performed by QIAGEN TissueLyser in 4 min high-speed (29 Hz) shaking steps. Mosquito DNA was extracted by Thermo Scientific GeneJET Gel Extraction Kit following manufacturer’s instructions. DNA was stored at −20 °C for polymerase chain reaction (PCR) analysis.

### Molecular detection of *kdr* alleles

Genotype analyses of knock-down resistance (*kdr*) mutations in the voltage-gated sodium channel (VGSC) gene (substitutions at codon 1014) were assessed in *Anopheles* mosquitoes using the protocol described in Syafruddin et al. [30]. Briefly amplification of a 300 bp segment of the voltage sodium-gated (VSG) channel gene flanking the 1014 position was performed using the primer pair Ag-F kdr (5′-GACCATGATCTGCCAAGATGGAAT-3′) and An-kdr-R2 (5′-GAGGATGAACCGAAATTGGAC-3′). The PCR mix was composed of 1 unit of Tfi DNA polymerase (Invitrogen™, Carlsbad, USA); 200 µM of dNTP mix (Invitrogen™) which corresponded to 200 µM of each dNTP; 1.5 mM of MgCl2 (Invitrogen™) and 400 µM of each primer. PCR was conducted in a total reaction volume of 50 µL (3 µL of DNA template and 47 µL of PCR mix) using the following amplification protocol: 5 min at 94 °C and 30 s, 45 °C and 1 min 30 s at 72 °C for 1 cycle and then 30 s at 94 °C, 30 s at 50 °C and 1 min at 72 °C for 29 cycles. Sequencing of the PCR product was performed by Macrogen™ (Seoul, South-Korea) using both primers. Each sequence was checked and cleaned manually using the Bioedit software version 7.1.9 [31]. Consensus sequence was generated for each specimen using the CAPS3 sequence assembly program [32]. and then aligned using the Clustal Omega multiple sequence alignment program [33].

### Data analysis

Mean mortality of each species across all surveys was determined for each village. The percentage mortality was adjusted by Abbott’s formula if the control mortality was between 5 to 20%. Results were interpreted according to WHO criteria: confirmed resistance (mortality below 90%), suspected resistance (mortality between 90 and 98%) and susceptible (mortality over 98%) [27]. Knockdown times, both 50% of tested population (KDT50) and 95% of tested population (KDT95), by exposure to three insecticides (DDT, deltamethrin, permethrin) in each study site were analysed separately by a log-time probit model using SPSS software (IBM^®^ SPSS^®^ Statistics version 23) (Table 2). Mortalities with and without synergists were provided with 95% confidence intervals.

**Table 2:** Knockdown time values (in minutes) of *Anopheles* mosquitoes according to sites of collection and insecticides

Site	Species	Insecticide	n	KDT_50_ [CI_95_] (min)	KDT_95_ [CI_95_] (min)
Pakla	*An. hyrcanus* s.l.	Deltamethrin 0.05%	11	39.1 [30.6–55.2]	220.7 [117.4–1106.2]
	*An. nivipes*	Deltamethrin 0.05%	17	8.4 [6.8–9.9]	16.6 [13.7–23.3]
	*An. maculatus* s.l.	Deltamethrin 0.05%	11	13.9 [11.2–16.1]	25.1 [20.9–35.3]
	*An.philippinesis*	Deltamethrin 0.05%	10	8.7 [6.5–10.7]	16.6 [13.1–27.2]
	*An. dirus* s.l.	Deltamethrin 0.05%	18	10.6 [9.1–11.8]	15.0 [13.2–21.5]
	*An. vagus*	Deltamethrin 0.05%	24	14.7 [8.8–20.1]	43.5 [29.9–102.6]
Talong	*An. hyrcanus* s.l.	Deltamethrin 0.05%	96	13.9 [12.3–15.5]	80.0 [68.3–97.6]
	*An. hyrcanus* s.l.	Permethrin 0.75%	100	16.2 [14.6–17.6]	78.6 [68.3–93.5]
	*An. hyrcanus* s.l.	DDT 4%	102	35.4 [31.0–40.9]	140.7 [102.4–235.1]
	*An. barbirostris* s.l.	Deltamethrin 0.05%	25	7.4 [5.6–9.1]	24.5 [20.0–32.8]
	*An. maculatus* s.l.	Deltamethrin 0.05%	19	14.7 [13.3–16.0]	21.9 [19.5–26.5]
Payaka	*An. maculatus* s.l.	Deltamethrin 0.05%	22	7.6 [6.5–8.8]	11.6 [9.9–15.6]
Nongmek	*An. hyrcanus* s.l.	Deltamethrin 0.05%	100	96.1 [77.7–131.4]	929.1 [512.9–2315.8]
	*An. hyrcanus* s.l.	Deltamethrin 0.05%	91	127.0 [89.1–263.9]	966.1 [400.3–6405.9]
	*An. hyrcanus* s.l.	Permethrin 0.75%	75	59.8 [49.8–77.6]	891.2 [465.7–2502.6]
	*An. hyrcanus* s.l.	DDT 4%	75	35.3 [31.6–39.6]	128.3 [98.8–190.4]
	*An. barbirostris* s.l.	Deltamethrin 0.05%	10	12.7 [10.0–15.3]	26.7 [21.5–38.8]
	*An. barbirostris* s.l.	Permethrin 0.75%	30	18.7 [17.0–20.2]	32.7 [29.5–37.6]
	*An. barbirostris* s.l.	DDT 4%	29	55.7 [51.9–61.7]	93.8 [79.4–126.2]
	*An. philippinesis*	Deltamethrin 0.05%	13	6.2 [4.6–7.7]	12.0 [9.4–21.7]
	*An. philippinesis*	Deltamethrin 0.05%	47	10.8 [9.8–11.6]	16.9 [15.4–19.7]
	*An. philippinesis*	Permethrin 0.75%	13	15.3 [13.1–17.4]	26.4 [22.5–35.0]
	*An. philippinesis*	DDT 4%	14	55.5 [49.4–68.9]	108.3 [81.6–218.1]
Sanghom	*An. hyrcanus* s.l.	Deltamethrin 0.05%	100	56.6 [49.5–67.5]	560.1 [355.8–1073.2]
	*An. hyrcanus* s.l.	Deltamethrin 0.05%	61	82.4 [60.1–158.2]	1018.5 [379.0–9977.9]
	*An. hyrcanus* s.l.	Permethrin 0.75%	100	32.4 [30.2–34.8]	140.0 [116.8–176.5]
	*An. hyrcanus* s.l.	Permethrin 0.75%	99	28.1 [25.0–31.3]	146.6 [112.1–216.1]
	*An. hyrcanus* s.l.	DDT 4%	100	60.1 [50.6–78.4]	274.7 [170.6–648.6]
	*An. barbirostris* s.l.	Deltamethrin 0.05%	50	11.7 [9.9–13.4]	24.2 [20.7–30.4]
	*An. barbirostris* s.l.	Permethrin 0.75%	12	16.1 [13.7–18.4]	27.7 [23.6–36.8]
	*An. barbirostris* s.l.	Permethrin 0.75%	25	16.1 [13.3–18.7]	32.3 [27.0–43.1]
	*An. barbirostris* s.l.	DDT 4%	12	60.5 [46.5–108.1]	290.6 [143.4–2066.4]
	*An. barbirostris* s.l.	DDT 4%	37	58.9 [55.6–64.7]	89.3 [77.5–116.1]
	*An. philippinesis*	Deltamethrin 0.05%	10	9.2 [6.7–11.1]	15.0 [12.3–24.9]
	*An. philippinesis*	Deltamethrin 0.05%	30	8.7 [7.5–9.6]	13.0 [11.6–16.0]
	*An. philippinesis*	Permethrin 0.75%	30	14.1 [12.6–15.6]	27.5 [24.3–32.5]
Nachalauy	*An. hyrcanus* s.l.	Deltamethrin 0.05%	73	48.9 [44.4–55.0]	218.1 [163.8–326.5]
	*An. hyrcanus* s.l.	Permethrin 0.75%	75	38.7 [33.7–45.8]	539.7 [318.5–1206.6]
	*An. hyrcanus* s.l.	DDT 4%	75	39.7 [32.5–51.4]	210.5 [125.7–587.4]
	*An. barbirostris* s.l.	Deltamethrin 0.05%	75	9.4 [8.6–10.1]	18.6 [16.9–21.1]
Chong Ta Ou	*An. barbirostris* s.l.	Deltamethrin 0.05%	10	22.0 [19.2–24.5]	32.9 [28.9–42.1]
	*An. nivipes*	Deltamethrin 0.05%	7	12.0 [8.8–14.6]	18.7 [15.2–38.7]
	*An. dirus* s.l.	Deltamethrin 0.05%	16	9.8 [7.7–11.7]	23.9 [19.4–32.9]
	*An. karwari*	Deltamethrin 0.05%	8	17.3 [15.2–19.5]	21.2 [19.0–31.1]

*KDT*_*50*_ estimated knockdown time for 50% of the population, *KDT*_*95*_ estimated knockdown time for 95% of the population, *CI*_*95*_ 95% confidence intervals

## Results

### Insecticide bioassays

**Fig. 2:**
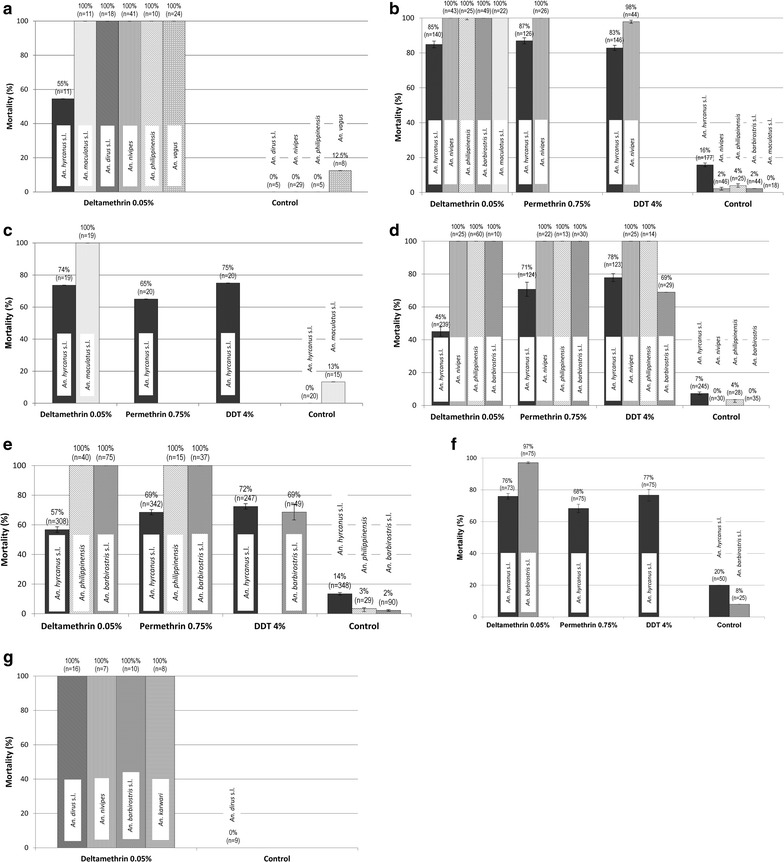
Insecticide susceptibility status of *Anopheles* mosquito species in **a** Ban Pakla, **b** Ban Talong, **c** Ban Payaka, **d** Ban Nongmek, **e** Ban Sanghom, **f** Nachalauy, **g** Chong Ta Ou. *Error bars* show confidence intervals

Results of bioassays are shown in the Fig. 2a–f. The most abundant species used for bioassays was *An. hyrcanus* s.l. with a total of 2088 specimens phenotyped in six collection sites. This species was tested against three insecticides (DDT; n = 611, deltamethrin; n = 790 and permethrin; n = 687) and was resistant to all three insecticides in all sites (mortality range from 45 to 87%). In Ban Pakla, *An. maculatus* s.l., *An. dirus* s.l., *An. nivipes*, *An. philippinensis* and *An. vagus* were fully susceptible to deltamethrin (mortality = 100%). In Ban Talong, bioassays results showed that all *Anopheles* species, except *An. hyrcanus* s.l. were susceptible to the insecticides tested. In Ban Payaka, *An. maculatus* s.l. was fully susceptible to DDT (100%). In Ban Nongmek, resistance to DDT was found in *An. barbirostris* s.l. with 69% mortality (n = 29), while *An. nivipes* (deltamethrin; n = 25, permethrin; n = 22, DDT; n = 25), and *An. philippinensis* (deltamethrin; n = 60, permethrin; n = 13, DDT; n = 14) were fully susceptible to the three insecticides (mortality = 100%). In Sanghom village, suspected resistance to DDT was found in *An. barbirostris* s.l., whereas *An. philippinensis* was susceptible to deltamethrin and permethrin. In Nachaluay, suspected resistance to deltamethrin was found in *An. barbirostris* s.l. (97%). In Chong Ta Ou, *An. dirus* s.l. (n = 16), *An. nivipes* (n = 7), *An. barbirostris* s.l. (n = 10), and *An. karwari* (n = 8) were susceptible to deltamethrin (mortality = 100%).

The range of KDT50 (estimated by the software) in all *Anopheles* tested was 35–61 min for DDT, 6.2–96.1 min for deltamethrin and 14.1–59.8 min for permethrin. The range of KDT95 was 11.6–1018.5 min for DDT, 89.3–290.6 min for deltamethrin and 26.4–891.2 min for permethrin. The highest KDT values were found in *An. hyrcanus* s.l. for deltamethrin (KDT50 = 127 min, KDT95 = 1018.5 min) and permethrin (KDT50 = 59.8 min, KDT95 = 891.2 min) and in *An. barbirostris* s.l. for DDT (KDT50 = 60.5 min, KDT95 = 290.6 min), respectively (Table 2).

### Synergist bioassays

**Fig. 3:**
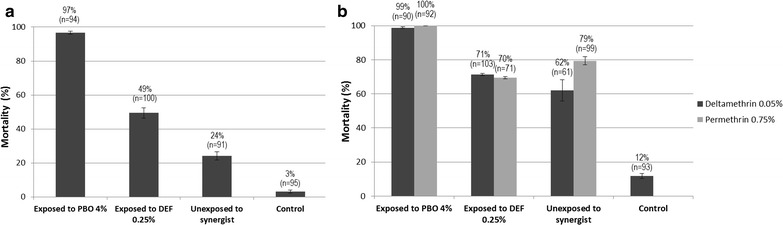
Efficacy of deltamethrin on *An. hyrcanus* s.l. from **a** Ban Nongmek, **b** Ban Sanghom with and without pre-exposure to the synergists PBO 4% and DEF 0.25%) (*Error bars* are confidence intervals)

Synergist bioassays were carried out with *An. hyrcanus* s.l. only because this species was resistant to all insecticides and adequate samples could be obtained for the test. In Ban Nongmek, pre-exposure of *An. hyrcanus* s.l. to synergists (PBO 4% and DEF 0.25%) significantly increased the insecticidal activity of deltamethrin; mortality shifted from 24% for deltamethrin alone to 49% and 97% for deltamethrin + DEF and deltamethrin + PBO respectively (Fig. 3a). A similar finding was observed in Ban Sanghom; mortality in *An. hyrcanus* increased from 79% for permethrin alone to 100% when pre-exposed to PBO and from 62% for deltamethrin alone to 99% when pre-exposed to PBO (Fig. 3b).

### *kdr* detection

**Fig. 4:**
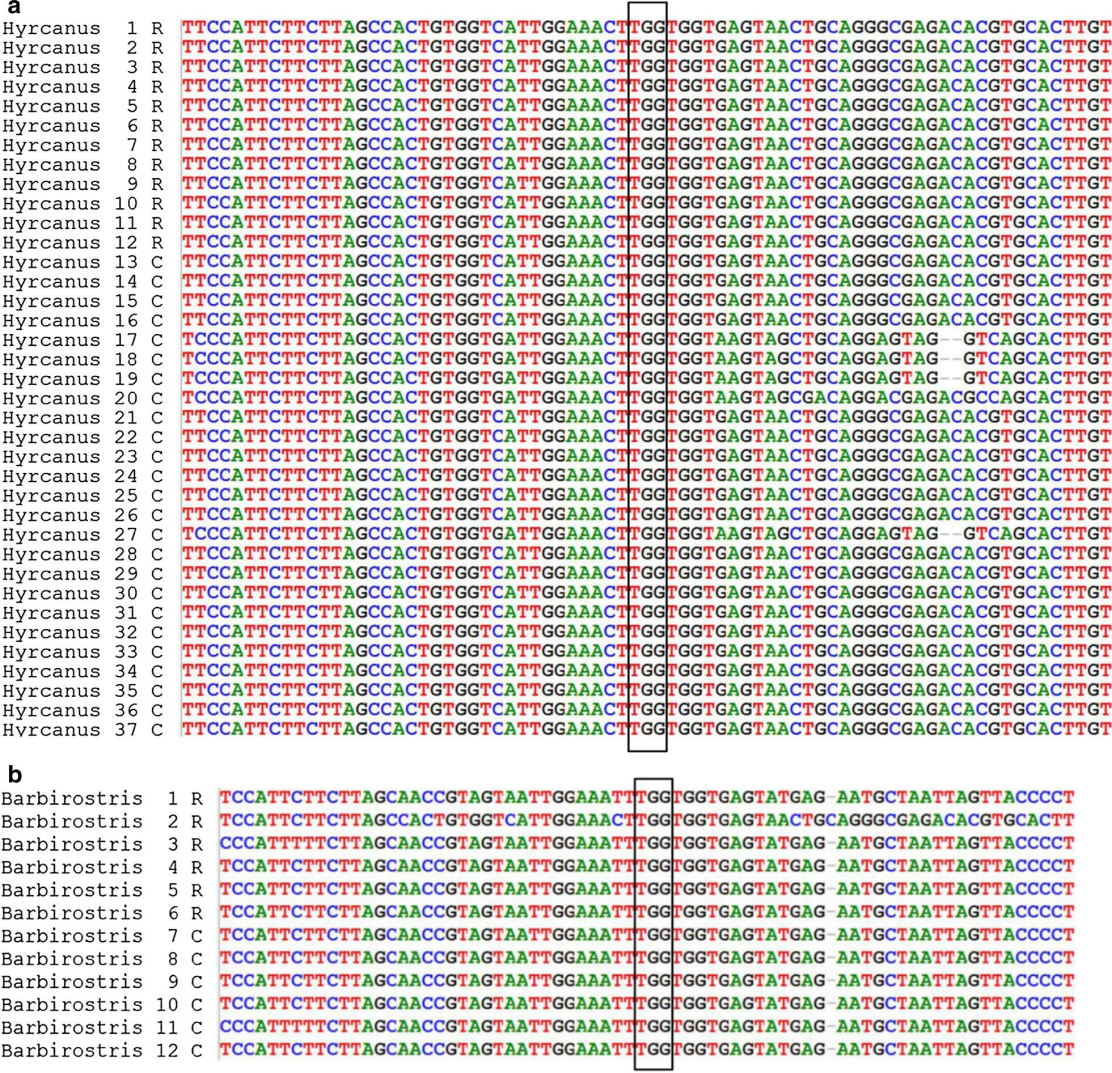
DNA sequence of voltage-gated sodium channel gene fragment from **a** *An. hyrcanus* s.l. and **b** *An. barbirostris* s.l. The *black rectangle* shows the codon at 1014 position. TTG indicate the wildtype susceptible allele (Leucine). Neither *kdr* alleles 1014L (TTA and CTA) nor 1014F (TTT) were found

DNA sequencing of the VGSC gene fragment was performed on 48 amplicons representing two *Anopheles* species (*An. hyrcanus* s.l. and *An. barbirostris* s.l.). Twelve *An. hyrcanus* s.l. mosquitoes surviving to insecticides (deltamethrin n = 4, permethrin n = 4, DDT n = 1, DEF + deltamethrin n = 1, DEF + permethrin n = 1, PBO + deltamethrin n = 1), and 25 control samples were sequenced for the presence of single nucleotide change at position 1014. Similarly, six *An. barbirostris* s.l. surviving exposure to bioassays (DDT n = 5 and deltamethrin n = 1) and 6 control samples were sequenced. None of the sequenced specimens presented either the L1014F or L1014S substitutions in the VGSC gene (Fig. 4). Other mutations were identified but their link with insecticide resistance is unknown. Synonymous mutation at position V1010 (C/G), previously described by Singh et al. [16], was observed in five samples among 37 sequenced *An. hyrcanus* s.l. specimens. Most of the genetic variability was found in the intron upstream the amplified VGSC gene fragment.

A full dataset on insecticide resistance and vector bionomics of *Anopheles* mosquitoes collected from Ubon Ratchatani is available online [34, 35].

## Discussion

Malaria vector control in Thailand targeting indoor resting and biting mosquitoes has contributed to reduce malaria in the country [6]. However, the increase of insecticide resistance in *Anopheles* mosquitoes in the Mekong region may represent a growing challenge for malaria control and elimination in the future [36].

Chemical approaches to control malaria vectors have been used for several decades in Thailand, especially insecticides for indoor residual spraying. Historically, there have only been a few published reports on insecticides resistance of primary malaria vectors and little monitoring on the susceptibility status of these mosquitoes. Between 1990 and 1997, foci of DDT resistance was detected in *An. dirus* s.l., *An. minimus* s.l. and *An. maculatus* in the northern part of Thailand [37]. Resistance to permethrin was found in a population of *An. minimus* s.l. from northern Thailand, based, however, on a lower discriminative dose than is used today (0.25% vs. 0.75%) [5, 37]. *Anopheles maculatus* was shown to be resistant to methyl parathion, probably due to the use of organophosphates for controlling pests in fruit orchards in Chiang Mai province [38]. Between 2012 and 2013, deltamethrin, cyfluthrin, and malathion resistance has been reported in *An. minimus* s.l. in the same province [4]. It is possible that more studies would reveal more widespread insecticide resistance in primary malaria vectors in Thailand; however, it is more likely that the lack of reports probably reflect a general absence of resistance in these vectors due to their habitats and biting behaviours. Malaria mosquitoes are not strongly exposed to insecticides, as they are elsewhere (e.g. in Africa) and thus the selection pressure is relatively low, resulting in low observed resistance.

This study was carried out to investigate the status of insecticide resistance in potential malaria vector species located in northeastern Thailand where a malaria outbreak has been observed in recent years. Due to insufficient numbers of field-caught *Anopheles* mosquitoes collected, full bioassays (n ≥ 100 mosquitoes) could not be achieved for most species. This finding showed that the primary malaria vectors *An. dirus* s.l. and *An. maculatus* s.l. were susceptible to pyrethroids, which means that current malaria vector control strategies relying on this insecticide class are still adequate. *Anopheles dirus* s.l. is strongly associated with deep forest including forested foothills or forest fringes areas [39]. Most of the *An. dirus* s.l. tested in bioassays were collected from Ban Pakla and Chong Ta Ou Thai border control station, located in a forested area. The absence of insecticide resistance in *An. dirus* s.l. could be because of low insecticide resistance selection pressure since they were collected from sites close to forests with little impact of both agricultural and public health insecticides. *Anopheles maculatus* s.l. were collected from Ban Pakla, Ban Talong and Ban Payaka, which are remote villages with potentially low insecticide pressure.

Conversely, *Anopheles hyrcanus* s.l. showed resistance to all insecticides tested. This species has been reported as a vector of filariasis and malaria in several studies of South, Southeast and East Asian regions, a fact that is sometimes underestimated by researchers and control programmes [40–42]. Members of the Hyrcanus Group include at least eight species that have been reported in Thailand [26]. Adult females of the Hyrcanus Group exhibit overlapping morphological characteristics, which often lead to misidentification. *Anopheles nigerrimus*, *An. peditaeniatus* and *An. sinensis* are considered as suspected vectors of malaria in Thailand and they have been incriminated as vectors of *P. vivax* in China and Korea [43]. In this study, more detailed morphological identification on a sub-sample of *An. hyrcanus* group indicated that they could be *An. peditaeniatus*, which is a common species in this area [44]. However, polymorphism observed in the intron upstream *kdr* 1014 mutation of five individuals from the Hyrcanus group, suggests a genetic diversity within the species of the group. Indeed, these five individuals appear to be closer genetically to *An. sinensis* while the others are closer to *An. peditaeniatus*. The intra-species genetic diversity of the VGSC gene could be further used for molecular characterization of sibling species [45, 46]. *Anopheles peditaeniatus* has been designated as a potential locally important vector of *P. vivax* [47, 48]. The occurrence of insecticide resistance in *An. hyrcanus* s.l. may be due to the fact that this species breeds in rice paddies in Asia where it can be exposed continually to several classes of chemicals for agricultural pest control, such as organophosphates, carbamates, pyrethroids, and organochlorines [49]. Suspected resistance to deltamethrin in *An. barbirostris* was found in Nachaluay. Even if this result has to be taken with caution considering the low sample size, this finding is worrying because *An. barbirostris* has been incriminated in malaria transmission along the Thai–Cambodian [50] and Thai–Myanmar borders [51, 52]. Occurrence and spread of resistance to insecticides even in secondary vectors could potentially change the modalities of transmission, hence compromising malaria vector control efforts. Better understanding of the diversity, insecticide susceptibility status and other factors relating to host seeking preferences of secondary vectors is then essential to develop better insecticide resistance management strategies, and consequently to success in malaria control and elimination.

In the GMS, several mutations at codon 1014 of the *kdr* allele, such as L1014F, L1014S, and L1014C have been reported in many *Anopheles* species [15, 30, 53, 54]. The presence of a L1014S *kdr* mutation was observed in *An. sinensis* and *An. peditaeniatus* which belongs to the Hyrcanus group [15]. In this study, no *kdr* mutations were detected in insecticide-resistant mosquitoes belonging to *An. hyrcanus* s.l. and *An. barbirostris* s.l. The absence of the *kdr* mutation suggests that resistance may be caused by other mechanisms such as cuticle modifications or metabolic resistance. The fact that piperonyl butoxide (PBO) restored susceptibility to pyrethroid insecticides in *An. hyrcanus* s.l., suggests that monooxygenase enzymes probably play a major role in metabolic resistance. In contrast, the slight increase of pyrethroid mortality in presence of DEF suggest a less important role of esterases in the resistance phenotype. Further investigations are needed to identify the molecular basis of metabolic resistance in *Anopheles* vectors in the GMS.

Insecticide susceptibility bioassays were performed following the WHO guidelines for insecticide resistance monitoring in malaria vector mosquitoes. WHO recommends three kinds of mosquito samples in the assessment of susceptibility tests, including adult females derived from larval collections, the F1 progeny of wild-caught female mosquitoes and wild-caught females directly. Accordingly, susceptibility tests are recommended to be performed on non-blood fed females aged 3–5 days post emergence. In this study, it was not possible to collect a sufficient number of larvae due to difficulties to locate breeding sites. The effect of factors such as different age of mosquitoes and blood-feeding status may affect the bioassay results since only field-collected female mosquitoes were used in the bioassays. As temperature can influence the toxicity of insecticides and relative humidity can affect mosquito survival, bioassays were performed in a shaded area and tubes were placed in a container covered with a wet towel in sheltered location. However, under the field condition, temperatures and humidity can vary considerably depending on the environment, so these factors may have impacted on mosquito mortality in bioassay tests.

The relative role of insecticides from agriculture and vector control in the selection of insecticide resistance is difficult to address. In agricultural areas, intensive use of pesticides was suspected to select for multiple resistance in a broad range of malaria vector species [9, 38]. In Ubon Ratchathani, most people work in the agricultural sector, especially growing rice as an economic crop, which covers large areas. It is also one of largest chili growing regions in Thailand [55]. Pesticides are routinely used to prevent insect damage and enhance market quality. The common pesticide classes in use are organophosphates such as chlorpyrifos and profenofos and a variety of pyrethroids [56, 57]. Insecticide resistance is most likely a result of both vector control and the expansion and intensification of agriculture with associated pesticides [13, 38]. Much work has to be done to understand the impact of agricultural practices on insecticide resistance selection to implement resistance management strategies [13]. Collaboration between agricultural and public health sectors is required to develop effective integrated pest and vector management interventions. The Global Vector Control Response, adopted by the 70th World Health Assembly in May 2017 advocate increased intra- and intersectoral collaboration and action for sustaining vector control and contribute to better management of public health pesticides [58].

## Conclusions

This study showed that pyrethroid resistance is present in Ubon Ratchathani, but rather limited to secondary malaria vector species. Achieving universal coverage and proper use of LLIN for all people at risk of the disease is still a priority. However, conventional strategies (i.e. LLIN and IRS) may not be optimal to protect people in this region who often work or sleep outdoors at night (e.g. rubber plantation workers, forest goers, sleeping in field huts) and are being exposed to exophagic mosquito vectors [59]. Indeed a recent serological study conducted along the Thai-Myanmar border showed that human antibody response to malaria vector bites was not significantly different between users and non-users of ITNs, suggesting limited personal protection [60]. As malaria transmission is forest-related, these border villagers are at risk of malaria infection due to forest activities [61]. Alternative control tools (e.g. insecticide treated clothes, spatial repellents or treated hammocks, etc.) adapted to the situation of people’s activities may be more effective to reduce the malaria burden [4, 62]. The magnitude and causes of residual transmission in malaria hotspots located in forested border areas, rubber plantations, logging camps, etc. should be further monitored through a combination of entomological, social, and epidemiological surveys. Continued monitoring of insecticide susceptibility and generating complementary data on resistance intensity to measure potential changes in the strength of resistance to public health pesticides is essential to ensure early detection of insecticide resistance in malaria vectors in the region.

## Abbreviations

IRS: indoor residual spraying
ITNs: insecticide-treated nets
WHO: World Health Organization
HLC: Human Landing Catch
CBC: cow bait collection
DDT: dichlorodiphenyltrichloroethane
PBO: piperonyl butoxide
DEF: S,S,S-tributyl phosphorotrithioate
VGSC: voltage-gated sodium channel
PCR: polymerase chain reaction
Kdr: knockdown resistance
SPSS: Statistical Package for the Social Sciences
DNA: deoxyribonucleic acid
